# Disentangling Large- and Small-Scale Abiotic and Biotic Factors Shaping Soil Microbial Communities in an Alpine Cushion Plant System

**DOI:** 10.3389/fmicb.2020.00925

**Published:** 2020-05-25

**Authors:** Chenyue Wang, Richard Michalet, Ziyang Liu, Xingpei Jiang, Xiangtai Wang, Gaosen Zhang, Lizhe An, Shuyan Chen, Sa Xiao

**Affiliations:** ^1^Ministry of Education Key Laboratory of Cell Activities and Stress Adaptations, Lanzhou University, Lanzhou, China; ^2^Environnements et Paléoenvironnements Océaniques et Continentaux, University of Bordeaux, Bordeaux, France; ^3^Key Laboratory of Desert and Desertification, Northwest Institute of Eco-Environment and Resources, Chinese Academy of Sciences, Lanzhou, China; ^4^Key Laboratory of Extreme Environmental Microbial Resources and Engineering, Lanzhou, China

**Keywords:** cushion plant, phenotypes, facilitation, high-throughput sequencing, soil microbial community, scale

## Abstract

Microorganisms play a crucial role in biogeochemical cycles and ecosystem processes, but the key factors driving microbial community structure are poorly understood, particularly in alpine environments. In this study, we aim to disentangle the relative contribution of abiotic and biotic factors shaping bacterial and fungal community structure at large and small spatial and integration scales in an alpine system dominated by a stress-tolerant cushion species *Thylacospermum ceaspitosum*. These effects were assessed in two mountain ranges of northwest China and for two contrasting phenotypes of the cushion species inhabiting two different microtopographic positions. The large- and small-scale abiotic effects include the site and microhabitat effects, respectively, while the large- and small-scale biotic effects include the effects of cushion presence and cushion phenotype, respectively. Soil microbial communities were characterized by Illumina Miseq sequencing. Uni- and multivariate statistics were used to test the effects of abiotic and biotic factors at both scales. Results indicated that the site effect representing the soil pH and abiotic hydrothermal conditions mainly affected bacterial community structure, whereas fungal community structure was mainly affected by biotic factors with an equal contribution of cushion presence and cushion phenotype effects. Future studies should analyze the direct factors contributing to shaping microbial community structure in particular of the cushion phenotypes.

## Introduction

Microorganisms are ubiquitous and play key roles in biogeochemical cycles (Delgado-Baquerizo et al., 2016; Fierer, 2017). They also contribute to shaping plant community diversity and sustaining terrestrial ecosystem functions (van der Heijden et al., 2008; Bardgett and van der Putten, 2014). Microbial community structure (i.e., biomass, species richness, and composition) and ecological distribution are influenced by a variety of interacting abiotic and biotic factors (Wardle et al., 2004; Singh et al., 2009; Andrew et al., 2012; de Vries et al., 2012). These ecological filters are different from those of macro-organisms such as plants (Fierer and Jackson, 2006; Fierer et al., 2011; Singh et al., 2012). It has been proposed either that microbial distribution is influenced by geography (Caruso et al., 2011), or that microbes have a cosmopolitan distribution (Fenchel and Finlay, 2003). These alternative theories have stimulated important microbial research. Nevertheless, such researches are by far less common than for macro-organisms, likely due to the small size, unexpected high diversity of microbes and limited resolution of detection methods (Fierer and Jackson, 2006; Green and Bohannan, 2006; Schuster, 2008).

Biotic factors are known to also have a strong influence on the structure and ecological distribution of microbial communities, as direct factors (De Deyn et al., 2011; Barberán et al., 2015), or in interactions with abiotic factors (Yergeau et al., 2010; de Vries et al., 2012). In contrast to macro-organisms, microbes are major participants in material decomposition and, thus, are very likely to have different responses to the biotic and abiotic environment. Specifically, bacterial communities are highly influenced by abiotic factors, such as pH and soil moisture (Singh et al., 2009; Rousk et al., 2010; Bainard et al., 2016), whereas fungal communities are more influenced by biotic factors. This might be due to the strong dependence of the latter organisms on plant litter, rhizodeposition or the direct interactions with dominant plant species, such as pathogens and mycorrhiza (Sugiyama et al., 2008; Millard and Singh, 2009; Lange et al., 2014; Urbanová et al., 2015; Chen et al., 2017). Moreover, because of the differences in dispersion abilities, bacterial and fungal communities may show different biogeographic patterns, and respond to abiotic or biotic factors at different geographic scales (Yergeau et al., 2010; Roy et al., 2013; Li et al., 2017; Ma et al., 2017).

Concerning abiotic factors, soil bacterial communities appear to be more dependent on local soil variables, such as pH, C/N, and water content than on regional abiotic factors, such as climate (Fierer et al., 2011; Bahram et al., 2018). The contribution of local (centimeters to meters) and regional (kilometers to thousands of kilometers) (Bardgett and van der Putten, 2014) abiotic factors to fungal community structure and ecological distribution depend on the scale that is inquired. Climate factors or geographic distance showed a higher contribution to the variation of the fungal community at the regional scale, whereas edaphic variables contribute more at the local scale (Wu et al., 2013; Tedersoo et al., 2014; Chen et al., 2017). Concerning biotic factors (such as plant diversity and genotypes), more focus has been made on plant presence than intraspecific variation effects on soil microbial community (Eskelinen et al., 2009; Weber et al., 2015; Wang et al., 2016; Gao et al., 2017), although intraspecific variation in plant traits has been shown to also influence soil microbial communities (Schweitzer et al., 2008; Yao and Wu, 2010; Courty et al., 2011; Madritch and Lindroth, 2011; de Vries et al., 2012; İnceoğlu et al., 2012). However, compared to abiotic factors, to our knowledge, there has been no study exploring the relative importance of plant presence vs. phenotypic variation in the biotic factors driving soil bacterial and fungal communities. Recently, the effect of different genotypes of cushion plants on soil fungal community has been shown (Roy et al., 2018). In addition, although the relative roles of abiotic and biotic factors on microbial community have been studied at the individual plant level or the ecosystem level (Philippot et al., 2013; Tripathi et al., 2013; Wang et al., 2017), there has been no study assessing the relative importance of abiotic and biotic factors acting at different geographical and integration scales for shaping microbial community structure.

Cushion plants are common nurse plants found in alpine, sub-alpine, Arctic and sub-Arctic habitats (Cavieres and Badano, 2009; Aubert et al., 2014). Cushion plants have been shown to have positive effects on neighboring plants via temperature amelioration (Arroyo et al., 2003), water retention (Schöb et al., 2012) or increase in nutrient availability (Cavieres et al., 2005; Schöb et al., 2012; Roy et al., 2013). However, some cushion plants are also known for their negative effects on understory plants, in particular, those species or phenotypes having very tight canopies (Al Hayek et al., 2015a, b; Bonanomi et al., 2016; Jiang et al., 2018; Pistón et al., 2018). Additionally, in the alpine belt, the beginning of soil formation occurs at the habitats occupied by cushions and their influence on soil organic matter is crucial (Roy et al., 2013). Thus, the microhabitats covered by cushions are like islands embedded in a bare rock background. Their specific characteristics and large distribution in alpine ecosystems made them an ideal model to illustrate the effects of abiotic and biotic factors on soil microbial communities. Casanova-Katny et al. (2011) investigated the role of fungal root symbionts in the facilitative effects of the cushion plant *Azorella madreporica* on their associated plants. They found that the mycorrhization of three native plants was six times higher for individuals associated with *A. madreporica* than individuals outside cushions, and the number of spores also showed a similar trend. Roy et al. (2013) investigated in the French Alps by capillary-electrophoresis single-strand conformation polymorphism (CE-SSCP) the effects of the cushion plant *Silene acaulis* on the composition of bacterial and fungal communities in soils from calcareous and siliceous rocks. They found that cushion plants structured microbial communities and that this foundation effect was stronger in stressful, acidic and nutrient-limited environments on siliceous rocks. Moreover, bacterial community composition was more similar inside cushions compared to outside, due to the soil pH buffering by cushions. By comparison, genotyping of cushions showed a strong effect on fungal community composition, even when the topography and soil properties were considered (Roy et al., 2013, 2018).

Recent studies have documented that several alpine cushion plant species worldwide exhibited two distinct phenotypes, i.e., ‘tight’ cushions and ‘loose’ cushions, in relation to varying stress conditions induced by mesotopographic positions (Michalet et al., 2011, 2016; Al Hayek et al., 2014, 2015a,b; Jiang et al., 2018; Pistón et al., 2018). ‘Tight’ cushions with dense canopies and convex morphology are more frequent at habitats with infertile soils from convex mesotopography, whereas ‘loose’ cushions with a more open canopy and flat morphology are more frequent at habitats with more fertile soils from concave mesotopography. The former phenotypes have dominant negative effects on other plant species, whereas the latter ones have important facilitative effects (Michalet et al., 2011, 2016; Al Hayek et al., 2015a).

It has been shown that both varying environmental conditions and dominant plant genotypes may alter the effect of cushion plants on soil microbial communities (Roy et al., 2018). To expand on these findings, alpine cushion systems from western China dominated by two contrasting phenotypes of the cushion plant *Thylacospermum caespitosum* (Michalet et al., 2016; Jiang et al., 2018) were selected in order to assess the relative roles of large- and small-scale abiotic and biotic factors in shaping the structure of soil microbial communities. Large-scale abiotic effects (site effects) were assessed by sampling two sites in different regions and climates (Qilian and Tianshan Mountains). Thus, the site effects were related to the abiotic changes at regional scale (Lortie et al., 2004; Michalet et al., 2015). Small-scale abiotic effects (microhabitat effects) were assessed in two contrasting mesotopographic positions (unfertile convex and fertile concave) (Brooker et al., 2009; Michalet et al., 2015). Large-scale biotic factors were assessed sampling below cushions and in adjacent open areas (cushion presence/absence treatment), whereas small-scale biotic factors were assessed sampling at the microhabitats of two contrasting phenotypes of the cushions. We aim to answer the following questions: (1) Does the relative importance of abiotic and biotic factors vary between bacteria and fungi? (2) Does the relative importance of regional versus local scale abiotic factors vary between bacteria and fungi? (3) Does the relative importance of cushion presence versus cushion phenotype effects vary between bacteria and fungi? Our study has the potential to better understanding the role of facilitation for ecosystem functioning and soil microbial diversity in alpine environments.

## Materials and Methods

### Materials, Experimental Design, and Soil Sampling

The study was conducted in two alpine sites (Qilian and Tianshan Mountains) with contrasting climate conditions. The Qilian Mountain site (hereafter QL, 39.590°N, 96.433°E, and 3696 m elevation) is located in the Yanchiwan national nature reserve, in Gansu Province, close to the edge of Qaidam Basin. Annual precipitation is 167 mm and mean annual temperature −3.9°C. Tianshan-mountain field site (hereafter TS, 43.117°N, 86.821°E, and 3772 m elevation) is situated in Xinjiang Province, with 312 mm of annual precipitation and −7.2°C of mean annual temperature. Both sites were located in northwest China where the climate is a typically continental temperate climate with a maximum of rainfall during the summer season. However, the Qilian site is drier than the Tianshan site, likely due to more western oceanic influences reaching the latter but not the former (Jiang et al., 2018). In addition to the obvious differences of hydrothermal conditions, the variance of abiotic factors between these two sites were mainly reflected by soil pH. Meanwhile, the abiotic factor differences of microhabitat were mainly due to soil nutrient contents (total organic carbon and C/N) (Jiang et al., 2018, also Supplementary Figure S3).

**FIGURE 1:**
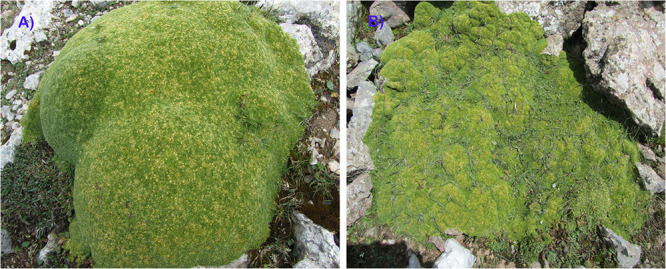
Different phenotypes of *Thylacospermum caespitosum* (Caryophyllaceae). **(A)**: tight phenotype; **(B)**: loose phenotype.

At Qilian and Tianshan sites, the plant communities are dominated by the alpine cushion plant *Thylacospermum ceaspitosum* (Caryophyllaceae), which is one of the most drought-tolerant alpine cushion species in the world, occurring between 3600 and 5900 m of elevation depending on latitudes (de Bello et al., 2011; Řeháková et al., 2015). In the previous study, two phenotypes of *T. caespitosum* have been observed in Ladakh (India) (Dvorský et al., 2013). Michalet et al. (2016) and Jiang et al. (2018) have shown that at our sites these two phenotypes occurred in contrasting habitats in relation to local mesotopographic variations. Tight dome-shaped phenotypes occur on convex topography on shallow drier soils, whereas loose flat phenotypes occur on concave topography with deeper and wetter soils (Figure 1), as described for other alpine cushion species (Michalet et al., 2011; Al Hayek et al., 2015a). Thus, these two contrasting microhabitats were used to assess at the two sites the contribution of the small-scale abiotic effects.

To assess the contribution of biotic factors to the diversity and composition of bacterial and fungal communities, we sampled the soils below cushions of the two phenotypes and in adjacent open areas with similar microhabitat conditions (i.e., with similar mesotopography and soil depth than the cushions) (Jiang et al., 2018). Three individual cushions of each phenotype of *T. caespitosum* were selected in late July of 2015 in each microhabitat for soil sampling. Then, four soil cores (0–10 cm deep) were obtained from both soils below the cushions and in open areas close to each cushion. All soil samples were sieved and the four soil cores form each replicate of each treatment were mixed thoroughly to produce one composite soil sample. A total of 24 soil samples [two sample sites × two phenotypes × two cushion treatments (i.e., inside and outside cushions) × three replicates] were transferred to sterile polythene bags, labeled and transported to the laboratory with cooler and then stored at −20°C for molecular analysis.

### Nucleic Acid Extraction and PCR Amplification

DNA of soil microbes was extracted by E.Z.N.A. Soil DNA Kit (Omega Bio-Tek, Inc., United States). The primer pair 338F-806R with barcodes was used to amplify bacterial V3–V4 regions fragments (Mori et al., 2014). ITS-5 and ITS-2 with barcodes were used to amplify fungal partial rRNA gene and ITS fragments (White et al., 1990). The total volume of PCR amplification is 20 μL, which contains 4 μL 5 × FastPfu Buffer, 2 μL 2.5 mM dNTPs, 0.4 μL FastPfu Polymerase (TransGen, CN), 0.8 μL 5 μM forward primer, 0.8 μL 5 μM reverse primer and 10 ng template DNA, respectively. PCR performed the following steps: (1) 95°C for 3 min; 27 cycles of 95°C for 30 s, 55°C for 30 s, 72°C for 45 s; then 72°C for 10 min and 2) 95°C for 3 min; 30 cycles of 95°C for 30 s, 58°C for 30 s; 72°C for 45 s; followed by 72°C for 10 min. In order to eliminate the bias induced by amplification, each sample had three duplicates and we mixed the product of the amplification from one sample. Then amplicons were extracted from 2% agarose gels and purified using the AxyPrep DNA Gel Extraction Kit (Axygen Biosciences, Union City, CA, United States) and quantified by QuantiFluor^TM^-ST (Promega, United States).

### Sequencing and Data Processing

The purified PCR products were subjected to paired-end sequencing by Illumina Miseq pair-end 300 at Majorbio BioPharm Technology Co., Ltd. (Shanghai, China). We used QIIME (version 1.9.1) for quality-filtering (Caporaso et al., 2010). Reads shorter than 50 bp or quality scores below 20 were removed and then paired reads that overlap longer than 10 bp were merged into one sequence according to overlap between pair-end reads. All merged sequences with any ambiguous bases or with any errors in either forward or reverse primer, or sequences with mismatch rate more than 0.2 among the overlapped region were removed. Chimeras were eliminated by using UCHIME (Edgar et al., 2011). Valid sequences without chimeras were subsequently clustered into different OTUs (Operational Taxonomic Units) by using UPARSE (version 7.1, Edgar, 2013) at 97% similarity cutoff. The taxonomy of each 16S rRNA and ITS OTUs were analyzed against the RDP (Cole et al., 2014) and UNITE (Nilsson et al., 2018) database at a default confidence threshold. Shannon diversity index was calculated by counting the number of observed OTUs through mothur (version v.1.35.1)^1^ (Schloss et al., 2009, 2011). In order to avoid the overestimation of diversity, the Shannon diversity index was calculated after eliminating singletons from all data. Relative abundances of different samples for bacteria and fungi on the phyla level or other main taxonomic groups were calculated. Moreover, we predicted the bacterial and fungal community functions profile with Faprotax (version 1.2.1, Louca et al., 2016) and FUNGuild (Nguyen et al., 2016), respectively. The ten most abundant OTUs of both bacterial and fungal communities were selected and assigned with the presumable specific function. The raw sequence reads have been submitted to the NCBI Sequence Read Archive repositories (BioProject ID: PRJNA516351).

### Statistical Analyses

All analyses were conducted with R (versions 3.4.3, R Development Core Team, 2015) using the packages “vegan” (Oksanen et al., 2017), “stats” (R Development Core Team, 2018) and “MASS” (Venables and Ripley, 2002). Figures were generated mainly by “ggplot2” package (Wickham, 2009) and “plotrix” (Lemon, 2006).

Three-way ANOVA using non-parametric tests was used to assess the effects of the site, habitat, cushion treatments and their interactions on Shannon diversity index and relative abundance of different taxonomic groups. Differences in microbial community composition among treatments were investigated with principal coordinate analysis (PCoA, “vegan” R package) based on Bray-Curtis dissimilarities using three-way non-parametric multivariate analysis of variance (NPMANOVA, “vegan” R package, Anderson, 2001) with 999 permutations. Variance partitioning analyses were conducted on the three-way ANOVA of microbial Shannon diversity and the three-way NPMANOVA of the microbial composition obtained from OTUs to quantify the relative contribution of each explanatory factor to soil microbial community variances (“stats” R package, “MASS” R package). Based on the tested abiotic factors (Jiang et al., 2018, Supplementary Figure S3), the site effect was used to quantify the large scale abiotic effects, which were mainly related to soil pH and abiotic hydrothermal conditions. The habitat and the habitat × site interaction effect were used to quantify the small scale abiotic effects (microhabitat effects) because different phenotypes and their paired-open microsites occurred in different local environmental conditions. Additionally, this microhabitat effects were mainly related to soil nutrition contents. The cushion and the site × cushion interaction effect was used to quantify the cushion presence effects (large-scale biotic effects), whereas the habitat × cushion and site × habitat × cushion interaction effects were used to quantify the cushion phenotypic effects (small-scale biotic effects) (Table 1).

**TABLE 1:** Abiotic and biotic factors at different scales.

Factors	Scales	Explanations
**Abiotic**		
Site	Site (large-scale)	Effects of this treatment are related to abiotic factor changes at regional scale, here, the effects are mainly due to the variance of soil pH and abiotic hydrothermal conditions.
Habitat Habitat × site	Microhabitat (small-scale)	Effects of these treatments are related to abiotic changes in meso-topography, here, the effects are mainly due to the variance of soil nutrients.
**Biotic**		
Cushion Cushion × site	Cushion presence	Effects of these treatments are relevant to cushion presence/absence (i.e., differences between cushion and open microhabitats).
Cushion × habitat cushion × habitat × site	Cushion phenotype	Effects of these treatments are relevant to cushion phenotypes (i.e., loose- and tight-cushions).

## Results

A total of 362,331 raw 16S rRNA gene sequences and 723,303 raw ITS sequences were obtained from the 24 soil samples collected in all treatments combinations. After quality filtering, we obtained a total of 324,709 and 369,086 valid sequences for bacteria and fungi, respectively. Valid sequences were assigned to a total of 2,440 OTUs for the bacteria and 1,457 OTUs for the fungi.

**FIGURE 2:**
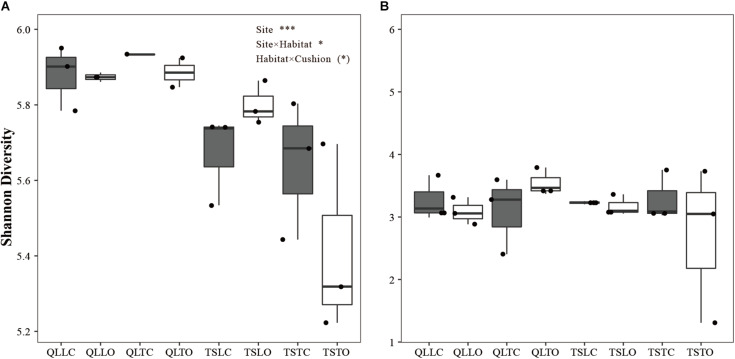
Mean (±SE) Shannon diversity index for **(A)** bacterial and **(B)** fungal communities from the soils of the loose and tight phenotypes of *Thylacospermum caespitosum* (black boxes) and their paired open plots (white boxes) in the two mountain ranges. The first three letters of QLL and QLT indicate loose and tight phenotypes in the Qilian mountains, respectively; TSL and TST indicate loose and tight phenotypes in the Tianshan mountains. Results of the effects of the Site, Habitat, Cushion treatments and their interactions are indicated above panel. (*)*P* < 0.1; *P < 0.05; ***P < 0.001.

There was a highly significant site effect on the Shannon index of bacterial communities, due to slightly higher diversity in the Qilian Mountains than in the Tianshan Mountains (*P_*S*__*ite*_* < 0.001, Figure 2A). There was also a weakly significant site × habitat interaction, due to lower bacterial diversity in the tight than in the loose habitat in the Tianshan Mountains only (*P_*Site* × *Habitat*_* < 0.05, Figure 2A). Finally, there was a marginally significant habitat × cushion interaction (*P_*Habitat* × *Cushion*_* < 0.1), due to a tendency in both mountain ranges for lower bacterial diversity in the open than below cushions in the tight cushion habitats and the converse in the loose cushion habitats. Altogether, these results show that the effect of abiotic factors on bacterial diversity, and in particular the site effect (related to soil pH and abiotic hydrothermal conditions) was significant and the effect was larger in effect size than other effects. In contrast, there was no significant effect of any abiotic or biotic factors on the Shannon index of fungal communities (Figure 2B).

**FIGURE 3:**
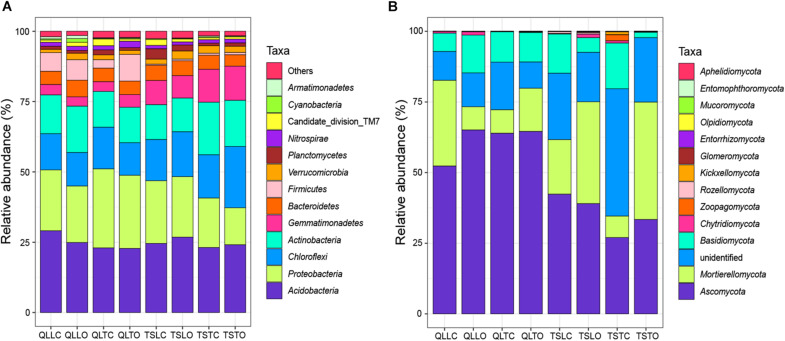
The stacked columns of **(A)** bacterial and **(B)** fungal community at the phylum level. “Others” of bacteria indicate the taxa with abundance below 1%. “Unidentified” of fungi indicate the OTUs without specific taxon.

**FIGURE 4:**
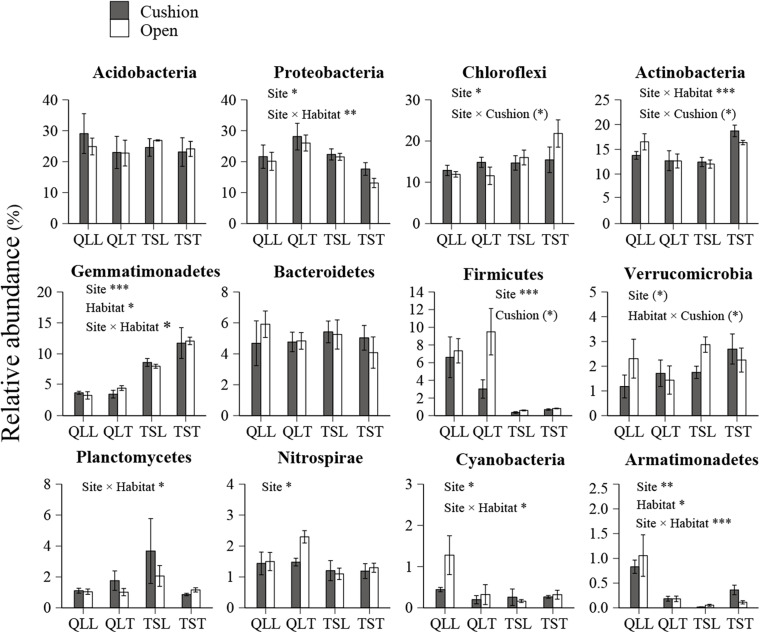
Mean (±SE) relative abundance of main bacterial taxonomic groups at the phylum level from the soils of the loose and tight phenotypes of *Thylacospermum caespitosum* (black bars) and their paired open plots (white bars) in the two mountain ranges. Legend of bars is the same as in Figure 2. Results of the effects of the Site, Habitat, Cushion treatments and their interactions are indicated above each panel. *P < 0.05; **P < 0.01; ***P < 0.001; (*)*P* < 0.1.

**FIGURE 5:**
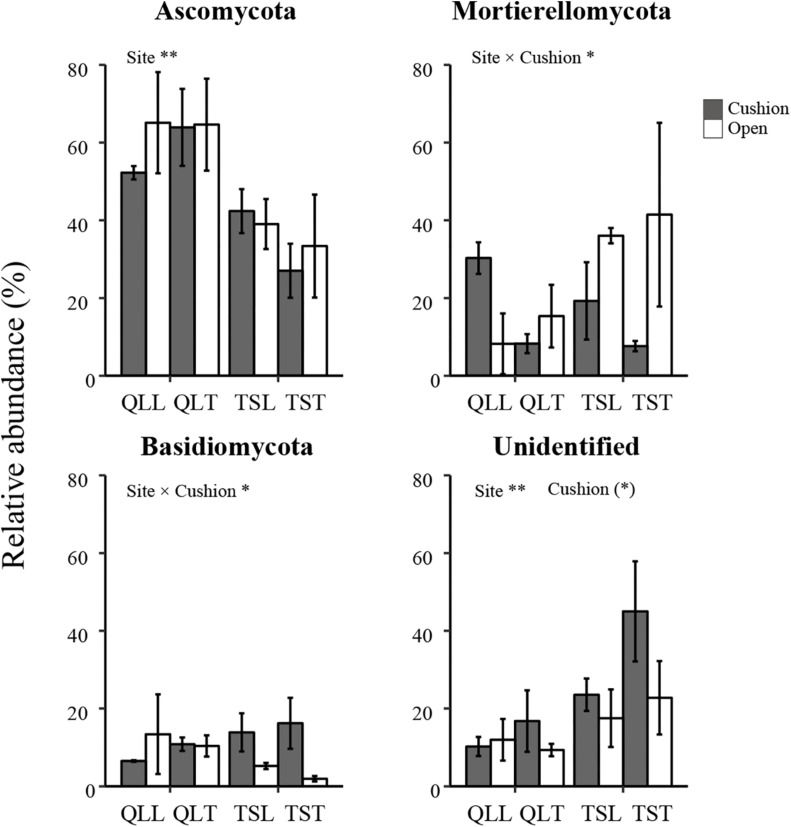
Mean (±SE) relative abundance of main fungal taxonomic groups at the phylum level from the soils of the loose and tight phenotypes of *Thylacospermum caespitosum* (black bars) and their paired open plots (white bars) in the two mountain ranges. Legend of bars is the same as in Figure 2. Results of the effects of the Site, Habitat, Cushion treatments and their interactions are indicated above each panel. *P < 0.05; **P < 0.01; (*)*P* < 0.1.

All the representative sequences with the highest abundance of each OTU were selected for the taxonomy assignment. The taxa with abundance below 1% were assigned to one group and named “others”. Finally, 14 phyla (39 orders) for bacteria and 13 phyla (22 orders) for fungi were examined (Figure 3). The relative abundances of *Proteobacteria, Chloroflexi, Gemmatimonadetes, Firmicutes, Verrucomicrobia, Nitrospirae, Cyanobacteria*, and *Armatimonadetes* were significantly affected by the site effect, with most of these phyla more abundant at the Qilian Mountains site, except *Chloroflexi* and *Gemmatimonadetes*, that were conversely more abundant at the Tianshan Mountains site (Figure 4). In addition, the relative abundances of *Proteobacteria, Actinobacteria, Gemmatimonadates, Planctomycetes, Cyanobacteria*, and *Armatimonadetes* were also significantly affected by the microhabitat effect, with specific variations across habitats at the two sites for each phylum (Figure 4). Only the relative abundances of three phyla (*Chloroflexi*, *Actinobacteria*, and *Firmicutes*) were marginally significantly affected by cushion presence and only the relative abundance of *Verrucomicrobia* was marginally significantly affected by cushion phenotype (Figure 4). For the fungi, the relative abundances of both *Ascomycota* and the entire unidentified taxa were significantly affected by the site effect, whereas the relative abundance of *Mortierellomycota, Basidiomycota* and the unidentified taxa were significantly affected by cushion presence (Figure 5). Also, *Ascomycota* is more abundant in Qilian than Tianshan while the unidentified taxa showed the opposite trend. Irrespective of the phenotype, *Mortierellomycota* is more abundant in open areas while *Basidiomycota* is more abundant below the cushions, but this is only true in the Tianshan mountain site.

**FIGURE 6:**
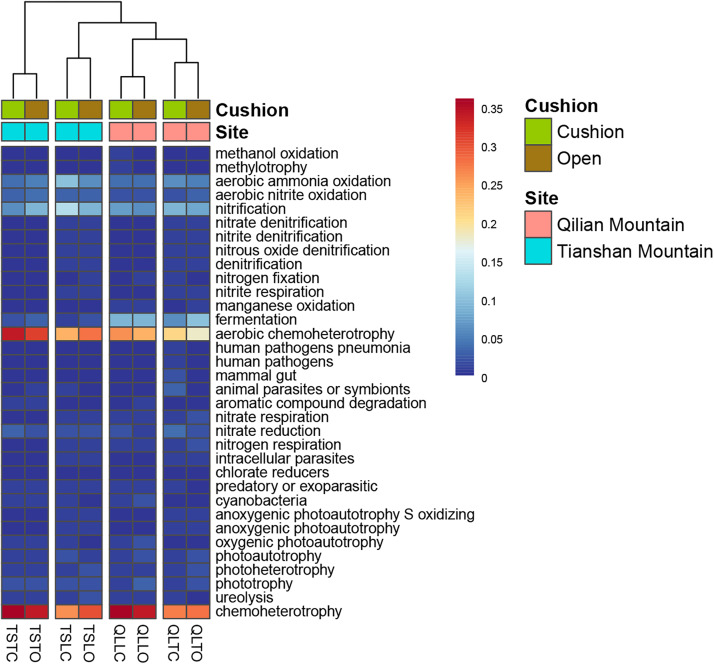
The functional profile of the bacterial community. The samples were clustered by the relative abundance of the assigned functional groups. The third letter of sample names (i.e., L and T) indicate the loose and tight phenotypes, respectively; the samples below cushions of the two phenotypes and in adjacent open areas which were labeled with green and brown colors, respectively.

**FIGURE 7:**
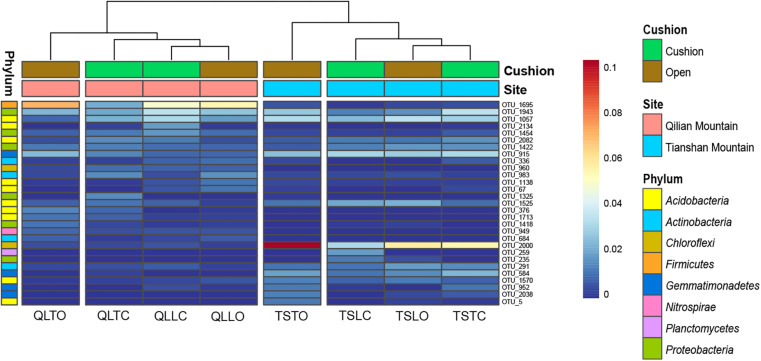
The 10 most abundant bacterial OTUs of each treatment. The color blocks on the left represent the taxa corresponding to each OTUs. The third letter of sample names (i.e., L and T) indicate the loose and tight phenotypes, respectively; the samples below cushions of the two phenotypes and in adjacent open areas which were labeled with green and brown colors, respectively.

**FIGURE 8:**
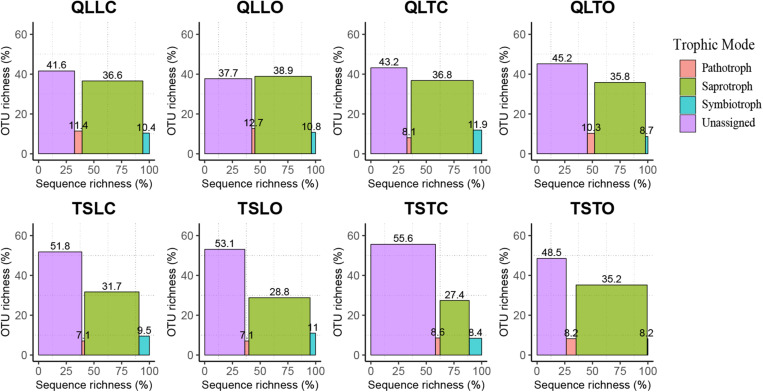
The functional profile of the fungal community predicted by FUNGuild. OTUs and sequences not assigned to trophic modes were placed into the unassigned groups. The proportion of sequence richness and proportion of OTU richness assigned to the trophic modes were represented by the *x*- and *y*-axis, respectively.

**FIGURE 9:**
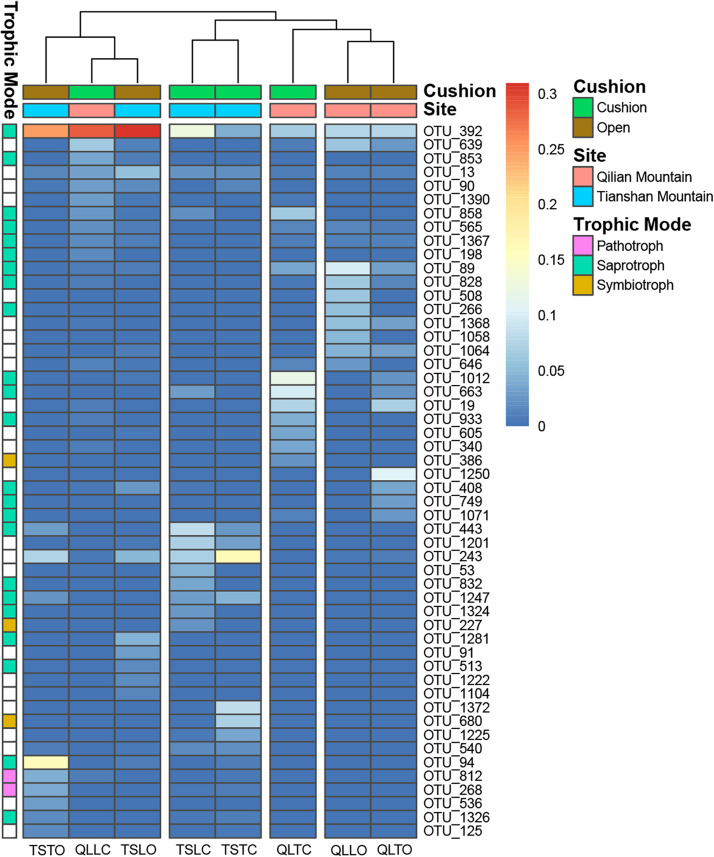
The 10 most abundant fungal OTUs of each treatment. The color blocks on the left represent the trophic modes assigned to each OTUs, and the white blocks of trophic mode indicate the taxa were not assigned functions.

The bacterial-community functions were predicted by Faprotax, and most of the bacterial taxa were not assigned the specific presumable functions, leading to a low relative abundance of the assigned functional groups. The aerobic chemo-heterotrophic groups and chemo-heterotrophic groups had the highest relative abundances, range from 0.2–0.35 and 0.28–0.35, respectively (Figure 6). Moreover, aerobic ammonia oxidation, nitrification and fermentation groups had the second-highest relative abundance, with 0.1–0.15 in values. The functional profile of the bacterial community was similar within the same habitat. The samples were clustered into four groups according to the sampling plots (Figure 6). The site and microhabitat effects and the cushion presence significantly influenced the presumable functions (Supplementary Figure S1). The ten most abundant bacterial OTUs of each treatment were selected to explore their functions, but only two OTUs (OTU1943 and OTU1418) were assigned the presumable function (aerobic- chemoheterotrophy and nitrite-denitrification, respectively). The result illustrated that the bacterial dominant OTUs were mainly assigned to *Acidobacteria*, *Proteobacteria, Gemmatimonadetes* and *Actinobacteria* (Figure 7). In particular, OTU2000 (*Chloroflexi*) showed higher relative abundance at tight-cushion habitat and loose-cushion paired open plots of Tianshan. Additionally, OTU1695 (*Firmicutes*) showed higher relative abundance at open plots of Qilian regardless of the phenotypes (Figure 7). Most of the fungal taxa were also not assigned presumable functions by the current FUNGuild analysis. A large number of the taxa with the presumable functions belonged to saprotroph, as the result showed high relative abundance for both OTUs and sequences (i.e., the area of each green bar, Figure 8). Moreover, only the site effect showed a marginally significant effect on the fungal functional groups (Supplementary Figure S2). The ten most abundant fungal OTUs were also selected to explore their functions, most of them belonging to saprotrophic modes which are consistent with the entire fungal community (Figure 9). The fungal trophic modes of dominant OTUs were similar, regardless of the phenotypes of the cushions, below cushions at the Tianshan site or at open plots of both the Qilian and Tianshan sites.

**FIGURE 10:**
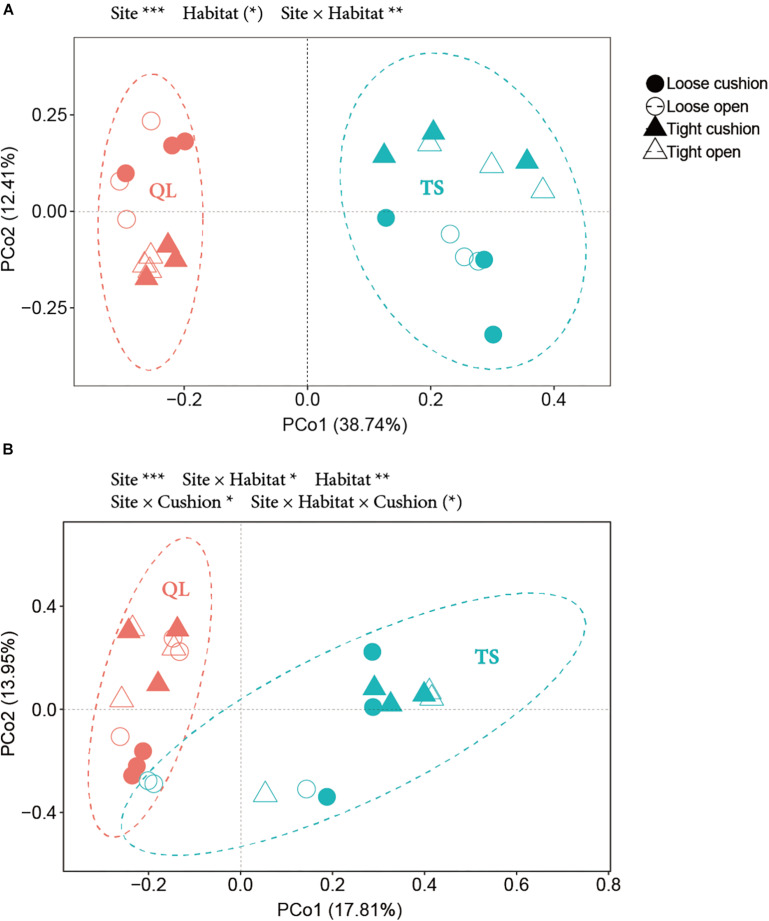
PCoA (Principal coordinate analysis) based on OTUs data from **(A)** bacteria and **(B)** fungi. Solid and hollow shapes indicate cushion and non-cushion plots, respectively. Circles and triangles indicate loose and tight phenotypes, respectively. Pink and blue indicate Qilian (QL) and Tianshan (TS) mountains, respectively. Ellipses show the 95% confidence intervals of the samples from each site. Results of treatment effects in the NPMANOVA are given on the top of each panel: * *P* < 0.05; ** *P* < 0.01; *** *P* < 0.001; (*) *P* < 0.1.

The results of the PCoA showed that the primary source of variation in OTUs composition was, for both bacterial and fungal communities, the large-scale abiotic effect, with always TS plots and QL plots separated along the first axis (highly significant site effect in both Figures 10A,B, both *P_*Site*_* < *0.001*). The second source of variation was for both community types the small-scale abiotic effects, with-in both cases contrasting positions along axis 2 for the concave and convex habitats depending on sites (significant site × habitat interactions in both Figures 10A,B, *P_*Site* × *Habita*_*_*t*_ < 0.01 for A, *P_*Site* × *Habitat*_* < 0.05 for B). However, only the fungal community was also affected by cushion presence and cushion phenotype effects (*P_*Site* × *Cushion*_* < 0.05, *P_*Site* × *Habitat* × *Cushion*_* < 0.1) (Figure 10B and Supplementary Table S2).

**FIGURE 11:**
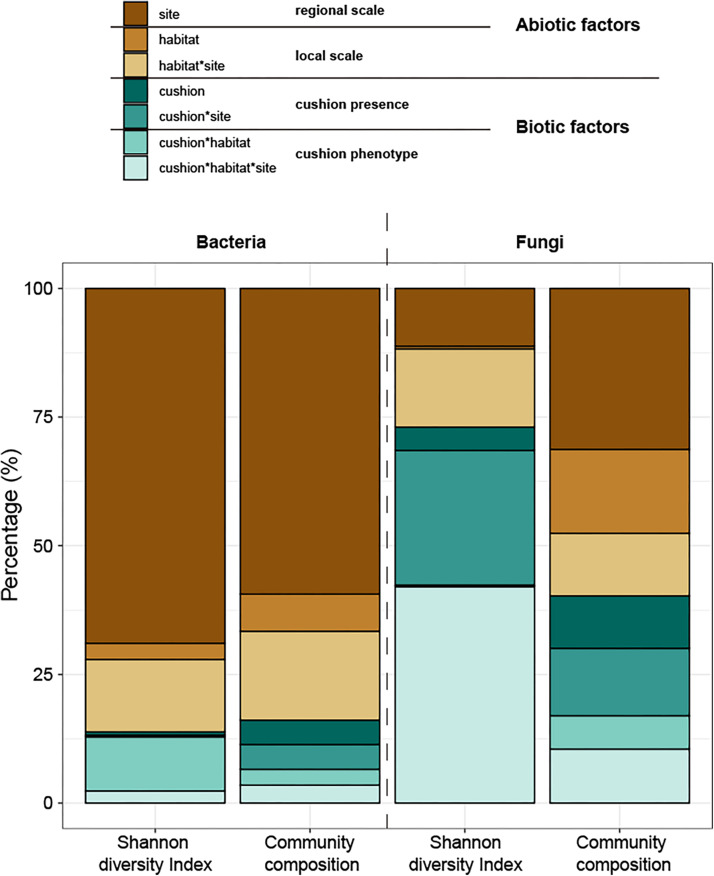
Variance partitioning of the different treatment effects and their interaction on the Shannon diversity indexes and composition of microbial communities. The colors of brown series indicate abiotic effects (regional and local abiotic factors); the colors of the green series indicate biotic effects (cushion presence and cushion phenotypes). Each color indicates the specific factor as shown in the legend on the top of the panel.

The total amounts of variation explained by the treatments were 73.8% and 20.5% for bacterial and fungal diversity, respectively and 62.3 and 45.0% for bacterial and fungal composition, respectively. Variance partitioning showed that bacterial diversity was primarily (69%) driven by the site effect, and only weakly (17%) by the microhabitat effects, whereas both cushion presence and their phenotypes had small effects (1 and 13%, respectively) (Figure 11). In contrast, fungal diversity was strongly driven by biotic factors with similar contributions of cushion presence (31%) and cushion phenotype effects (42%). Bacterial community composition was also primarily driven by the site effect (59%), with weak microhabitat effect (24%), whereas cushion-presence effects (10%) had a stronger contribution than the phenotypic effects (7%). Meanwhile, abiotic and biotic factors similarly contributed to fungal community composition (59 and 41%, respectively, Figure 11 and Supplementary Table S1).

## Discussion

Our study aimed at disentangling the effects of large- and small-scale abiotic and biotic factors in shaping soil microbial communities. Results of PCoA and variance partitioning consistently showed that the contribution of abiotic and biotic factors and of large and small-scale effects strongly differed among micro-organisms inhabiting similar environmental conditions in specific plant communities. Abiotic factors had a much higher influence than biotic factors for bacterial communities and the converse was observed for fungal communities, in particular for diversity. The site effect had a much higher influence on both bacterial diversity and composition than the microhabitat effect. But when the tested abiotic factors were considered (Supplementary Figure S3), the site effect was mainly related to soil pH and abiotic hydrothermal conditions, and the microhabitat effect was mainly related to soil nutrition contents (STOC and C/N). For biotic factors, both cushion presence and cushion phenotypes had a similar contribution to the composition of both community types, whereas the effects of cushion phenotypes had a much higher contribution to bacterial diversity than cushion presence.

### The Relative Contribution of Abiotic and Biotic Factors

Bacterial community structure was mainly driven by abiotic factors, in particular the site effect which was probably related to environmental filtering rather than dispersal limitation effects (Hanson et al., 2012; Xiao et al., 2018). The bacterial dominant OTUs showed convergence in the same site. Fungal dominant OTUs also showed the convergence except for fungal community inside the loose cushion of Qilian. Furthermore, previous studies documented the high dependency of bacterial community to abiotic factors and in particular pH (Lauber et al., 2009; Andrew et al., 2012; Shen et al., 2013). The result of this research also supported such conclusions, due to the site effect was mainly related to soil pH and the hydrothermal conditions. Additionally, other important direct factors for bacterial life generally covary with pH, and especially salinity and organic matter content and quality (Lozupone and Knight, 2007; Singh et al., 2009; Ma et al., 2017; Weil and Brady, 2017; Bahram et al., 2018). Soil pH is also relevant to the precipitation on a global scale (Lladó et al., 2017). This probably also explained the strong site effect in this study. In contrast, fungal community structure was mainly affected by biotic factors, which is consistent with previous studies showing that fungal community structure is related to plant diversity (Prober et al., 2014; Chen et al., 2018) and genetic variation (Roy et al., 2018). A recent research showed that the fungal diversity was also determined by the net primary productivity (Liu et al., 2020). These are mostly due to the important impacts of plants on soil fungal communities through root exudates (Broeckling et al., 2007; Zinger et al., 2011; Prober et al., 2014). Fungi can decompose high molecular substrates, such as cellulose and lignin (de Boer et al., 2005). This tight relationship might also be due to direct interactions between fungi and plants (Millard and Singh, 2009; Peay et al., 2013). Especially, mycorrhizal fungi and fungal plant pathogens usually show close correlations with plants (Berg and Smalla, 2009; Martínez-García and Pugnaire, 2011; Hiiesalu et al., 2014).

### The Relative Contribution of Site and Microhabitat Effects

We found that the site effect had a much higher influence on both diversity and composition than the microhabitat effect, especially for bacteria. However, the strong site effect was likely due to the variance of soil pH among others (such as precipitation and average summer temperature) (Supplementary Figure S3). Thus, this result is consistent with most previous studies showing an dominant influence of local soil factors. For example, in their continental-scale assessment of macro-organisms and soil bacterial communities, Fierer and Jackson (2006) found that the common predictors of macro-organisms were not suitable for bacteria, which geographical distribution was more influenced by local soil factors (see also Dequiedt et al., 2011). In general, microbial communities have been shown to be more influenced by local than regional soil factors, as found by Chu et al. (2010) in the arctic. Roy et al. (2013) also explored soil microbial communities in the alpine cushion plants system. They found a dominant effect of local variation in soil factors in relation to bedrock types, in particular for fungal communities that are highly influenced by differences in nutrient availability induced by bedrock types. In the recent global investigation of topsoil communities, local environmental variables are also the dominant factors for bacterial communities, and in particular, pH having the strongest effect before other abiotic factors (Bahram et al., 2018). Compared to bacteria, soil and climate variables had similar effects on fungal community composition (Bahram et al., 2018). In contrast, Wang et al. (2015) and Andam et al. (2016) found that regional abiotic parameters highly contribute to variation in bacterial community structure. The assessment of 248 isolates of *Pseudomonas* from ten sites with molecular technologies at different resolutions, showed that genotypic composition was mainly driven by geographical distance (Cho and Tiedje, 2000). These contrasting results of the literature might be explained by the scale of inquiry, with the effects of geographical distance more likely to show up at global (continental) scales than at smaller scales (Martiny et al., 2006). We also suggest that the dominant influence of site over the microhabitat found in our study might be due to stronger differences in soil conditions existing between mountain ranges than between topographic positions within mountain ranges in this alpine system, as shown by Jiang et al. (2018) on the same alpine system (Supplementary Figure S3).

Carbon mineralization rate was found to be the direct factor driving the relative abundance of bacterial phyla in the study of Fierer et al. (2007) on forest soils. In addition, *Acidobacteria*, *Proteobacteria*, *Chloroflexi*, *Verrucomicrobia*, and *Actinobacteria* may split into two different nutritional types, i.e., oligotrophic or copiotrophic types. The relative abundance of oligotrophic taxa, such as *Acidobacteria* and *Verrucomicrobia*, decreased with accelerating carbon mineralization rate, while copiotrophic (*Proteobacteria* and *Actinobacteria*) abundance increased. In our study, these phyla responded differently to the abiotic factors of different scales. This shows that not all microbial taxa manifest similar biogeographical patterns (Fierer, 2008). In addition, *Chloroflexi* (OTU2000) was more abundant at open plots and under tight cushions of Tianshan. Previous research has documented the *Chloroflexi* are usually distributed in oligotrophic conditions (Yamada et al., 2005) and tight cushions occur more frequently at habitats with infertile soils. Similarly, *Firmicutes* (OTU1965) can tolerate extreme conditions (Hortal et al., 2013), and it had higher abundances at tight-cushion paired open plots.

### The Relative Contribution of Cushion Presence and Cushion Phenotypes Effects

Our results also indicate that both cushion presence and cushion phenotype effects contributed similarly to the composition of both community types, whereas for bacterial diversity we found a much higher contribution of cushion phenotypes than cushion presence. The lack of studies assessing the relative importance of the effects of plant presence and plant phenotypic effects on microbial communities impedes a straightforward comparison of our results to those of the literature. However, Li et al. (2018) found that bacterial and fungal communities responded differently to spruce phenotypes with leaf water content significantly influencing fungal community composition, but not bacterial communities. In contrast, Aira et al. (2010) found that the soils of two different genotypes of maize hosted different bacterial and fungal communities. Different biotic effects mainly result from contrasting root excretions (Cregger et al., 2018; Li et al., 2018; Zhalnina et al., 2018), and generally, saprotrophic bacteria show faster response than saprotrophic fungi, the latter needing readily accessible energy for proliferation (Cardon and Gage, 2006). In nitrogen-poor conditions dominated by nutrient conservative plants with low relative growth rate and low specific leaf area, fungi are relatively more abundant than bacteria due to the higher advantage of the former organisms to degrade low-quality plant litter (de Vries et al., 2012). The result of FUNGuild showed that the abundance of saprotrophic fungi was much higher than that of pathogenic and symbiotic fungi (Figure 8). Furthermore, most of the dominant OTUs belonged to saprotrophic fungi which might be due to the traits of the cushion species *Thylacospermum caespitosum.* The results of Faprotax illustrated that the abundance of aerobic-ammonia-oxidation bacteria and nitrifiers were slightly higher than that of other functional groups (Figure 6). In our study, cushion presence had a weak influence on the relative abundance of *Actinobacteria*, *Chloroflexi*, and *Firmicutes*. Since eutrophic soils with more root exudates are generally preferred by *Actinobacteria*, the observed variation in relative abundance may result from the indirect effects of cushions on soil nutrients. Only the relative abundance of *Verrucomicrobia* responded slightly to the cushion phenotype effect. This taxon is known for its oligotrophic life history (Bergmann et al., 2011) and a crucial role in biogeochemical cycles (Pol et al., 2007). In addition, Buckley and Schmidt (2001) showed that this taxon was particularly sensitive to soil water content, which is consistent with the contrasting effects on water content found by Michalet et al. (2017) in Lebanon for two different phenotypes of the alpine cushion plant *Onobrychis cornuta*.

## Conclusion

We showed that the variance in soil bacterial community structure was mainly driven by the site effect which due to the variance of soil pH and hydrothermal conditions, whereas variance in fungal community structure was mainly driven by biotic factors with an equal contribution of cushion presence and cushion phenotype effects. Future studies should explore the underlying mechanisms of direct and indirect effects of different cushion phenotypes on soil microbial communities. Integrating below-ground biota and plant–plant interactions in ecosystem studies will certainly improve our understanding of ecosystem functioning and community dynamics in natural systems.

## Data Availability Statement

The original contributions presented in the study are included in the article/Supplementary Material; further inquiries can be directed to the corresponding authors.

## Author Contributions

RM and CW planned and designed the research. ZL, XJ, and XW helped to analyze the data. CW conducted the experiments and wrote the manuscript. All authors were involved in revising the manuscript critically.

## Conflict of Interest

The authors declare that the research was conducted in the absence of any commercial or financial relationships that could be construed as a potential conflict of interest.

## Footnotes

1 http://www.mothur.org
