# Supplementary material for: Disentangling Large- and Small-Scale Abiotic and Biotic Factors Shaping Soil Microbial Communities in an Alpine Cushion Plant System
Source: Front Microbiol. 2020 May 25;11:925. doi: 10.3389/fmicb.2020.00925 (PMC7262953; doi:10.3389/fmicb.2020.00925)
Supplement: Supplementary file 1 [file Data_Sheet_1.docx]

Supplementary Information

**Disentangling Large- and Small-Scale Abiotic and Biotic Factors Shaping Soil Microbial Communities in an Alpine Cushion Plant System**

**Supplementary Table 1 Variance partitioning for Shannon indexes and community composition**

| Factors | Bacteria | | Fungi | |
| --- | --- | --- | --- | --- |
|  | Shannon diversity Index | Species composition | Shannon diversity Index | Species composition |
| **Abiotic** |  |  |  |  |
| site | 0.6893 *** | 0.5940 *** | 0.1122 | 0.3126 *** |
| habitat | 0.0316 | 0.0721 (*) | 0.0054 | 0.1630 ** |
| habitat × site | 0.1407 * | 0.1728 ** | 0.1518 | 0.1220 * |
| **Biotic** |  |  |  |  |
| cushion | 0.0062 | 0.0473 | 0.0451 | 0.1014 |
| cushion × site | 0.0034 | 0.0480 | 0.2620 | 0.1312 * |
| cushion × habitat | 0.1054 * | 0.0311 | 0.0026 | 0.0646 |
| cushion × habitat × site | 0.0234 | 0.0347 | 0.4209 | 0.1052 |

*, *P* < 0.05; **, *P* < 0.01; ***, *P* < 0.001; (*), *P* < 0.1. The site treatment is relevant of regional (large-scale) abiotic factors, whereas the habitat and habitat × site interaction are relevant of local (small-scale) abiotic factors. The cushion and cushion × site interaction are relevant of cushion presence (large-scale) biotic effects, whereas the cushion × habitat and cushion × habitat × site interactions are relevant of cushion phenotype (small-scale) biotic effects.

**Supplementary Table 2 The results of PERMANOVA**

| Factors | Bacteria | | Fungi | |
| --- | --- | --- | --- | --- |
|  | R^2^ | Pr (>F) | R^2^ | Pr (>F) |
| **Abiotic** |  |  |  |  |
| site | 0.3699 | 0.0010 *** | 0.1406 | 0.0010 *** |
| habitat | 0.0449 | 0.0739 (*) | 0.0733 | 0.0080 ** |
| habitat × site | 0.1076 | 0.0020 ** | 0.0549 | 0.0460 * |
| **Biotic** |  |  |  |  |
| cushion | 0.0295 | 0.2318 | 0.0456 | 0.1109 |
| cushion × site | 0.0299 | 0.2028 | 0.0590 | 0.0300* |
| cushion × habitat | 0.0193 | 0.5065 | 0.0290 | 0.6713 |
| cushion × habitat × site | 0.0216 | 0.4046 | 0.0473 | 0.0889 (*) |

*, *P* < 0.05; **, *P* < 0.01; ***, *P* < 0.001; (*), *P* < 0.1. The factors in this table are in accordance with the Supplementary Table 1.

**Fig S1.** PCoA (Principal coordinate analysis) based on the relative abundance of the bacterial taxa with **the** functional assignment. QL and TS indicate Qilian and Tianshan. Solid and hollow shapes indicate cushions and open plots, respectively. Circles and triangles indicate the loose and tight phenotypes, respectively. Ellipses show the 95% confidence intervals of the samples from each site. Results of treatment effects in the NPMANOVA are given on the top of the panel: *, *P* < 0.05; **, *P* < 0.01; ***, *P* < 0.001; (*), *P* < 0.1.

**Fig S2.** PCoA (Principal coordinate analysis) based on the relative abundance of the fungal taxa with trophic mode assignment. QL and TS indicate Qilian and Tianshan. Solid and hollow shapes indicate cushions and open plots, respectively. Circles and triangles indicate the loose and tight phenotypes, respectively. Ellipses show the 95% confidence intervals of the samples from each site. Results of treatment effects in the NPMANOVA are given on the top of the panel: *, *P* < 0.05; **, *P* < 0.01; ***, *P* < 0.001; (*), *P* < 0.1.

**Fig S3**. Principal component analysis (PCA) on abiotic factors (SWC (%), soil water content; STOC (mg·g ^-1^), soil total organic carbon content; STP (mg·g^-1^), soil total phosphorus content; C/N, the ratio of total organic carbon content and total nitrogen content; pH; Preci (mm), average summer precipitation; T (°C), average summer temperature; alt (m), altitude of Qilian (QL) and Tianshan (TS) Mountains. Solid and hollow shapes indicate cushions and open plots, respectively. Circles and triangles indicate the loose and tight phenotypes, respectively. Ellipses show the 95% confidence intervals of the samples from each site. Arrows give the contributions of each variable. Asterisks refer to the significance of non-parametric multivariate analysis of variance (NPMANOVA, permutation=1000): *, *P* < 0.05; **, *P* < 0.01; ***, *P* < 0.001; (*), *P* < 0.1.
