# Supplementary figures and images for: Disentangling Large- and Small-Scale Abiotic and Biotic Factors Shaping Soil Microbial Communities in an Alpine Cushion Plant System
Source: Front Microbiol. 2020 May 25;11:925. doi: 10.3389/fmicb.2020.00925 (PMC7262953; doi:10.3389/fmicb.2020.00925)

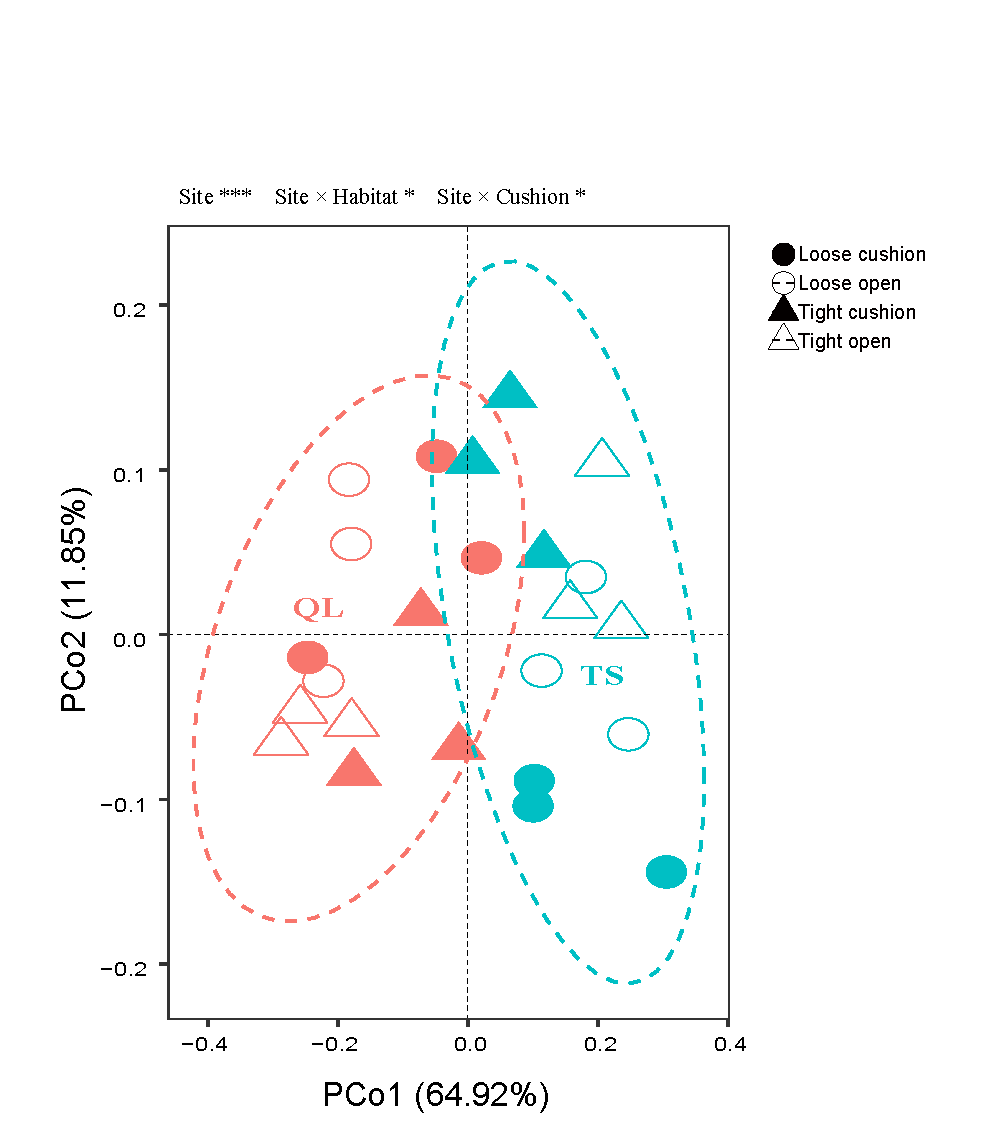

Supplement: Supplementary file 2 [file Image_1.tiff]

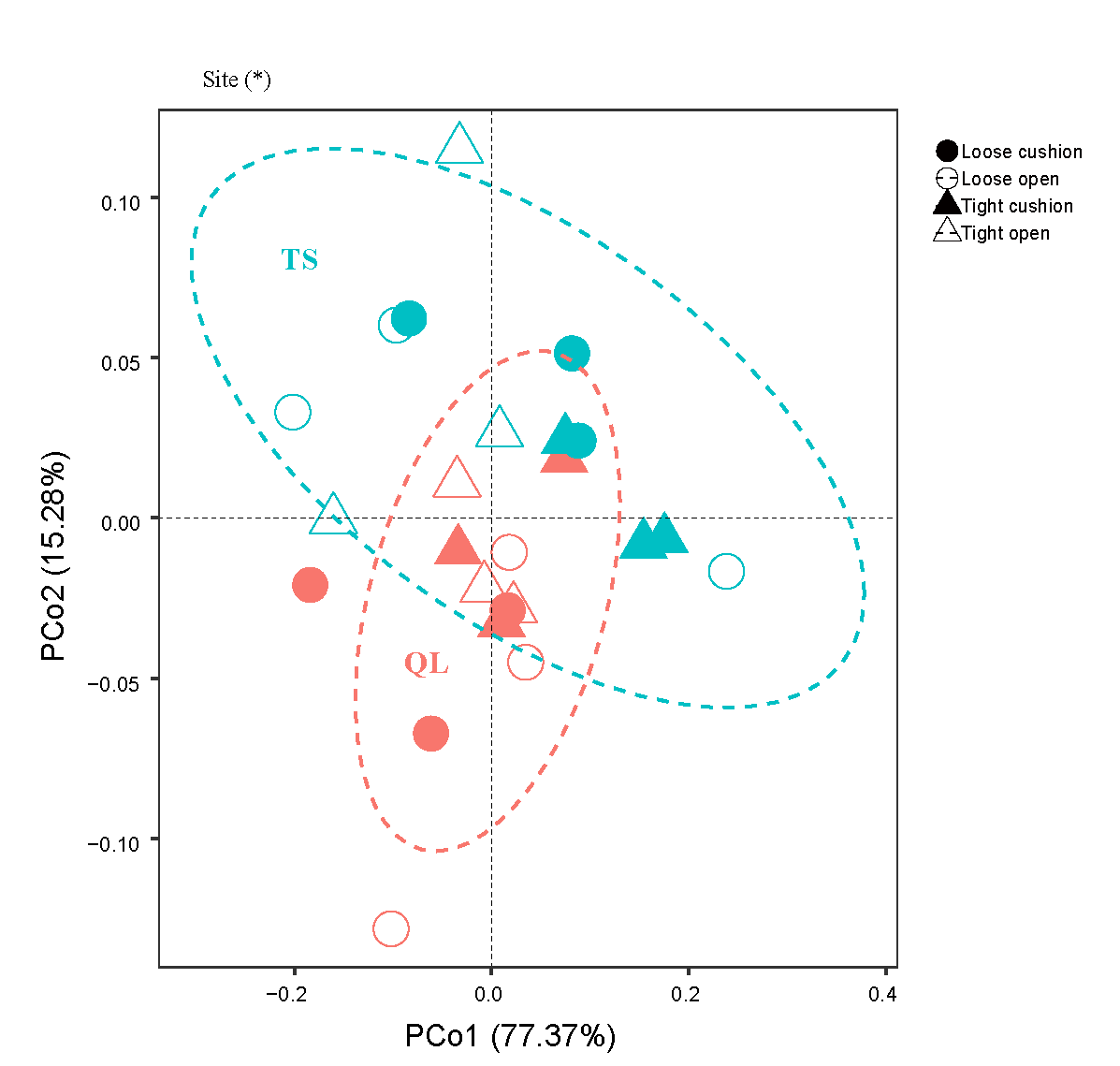

Supplement: Supplementary file 3 [file Image_2.tif]

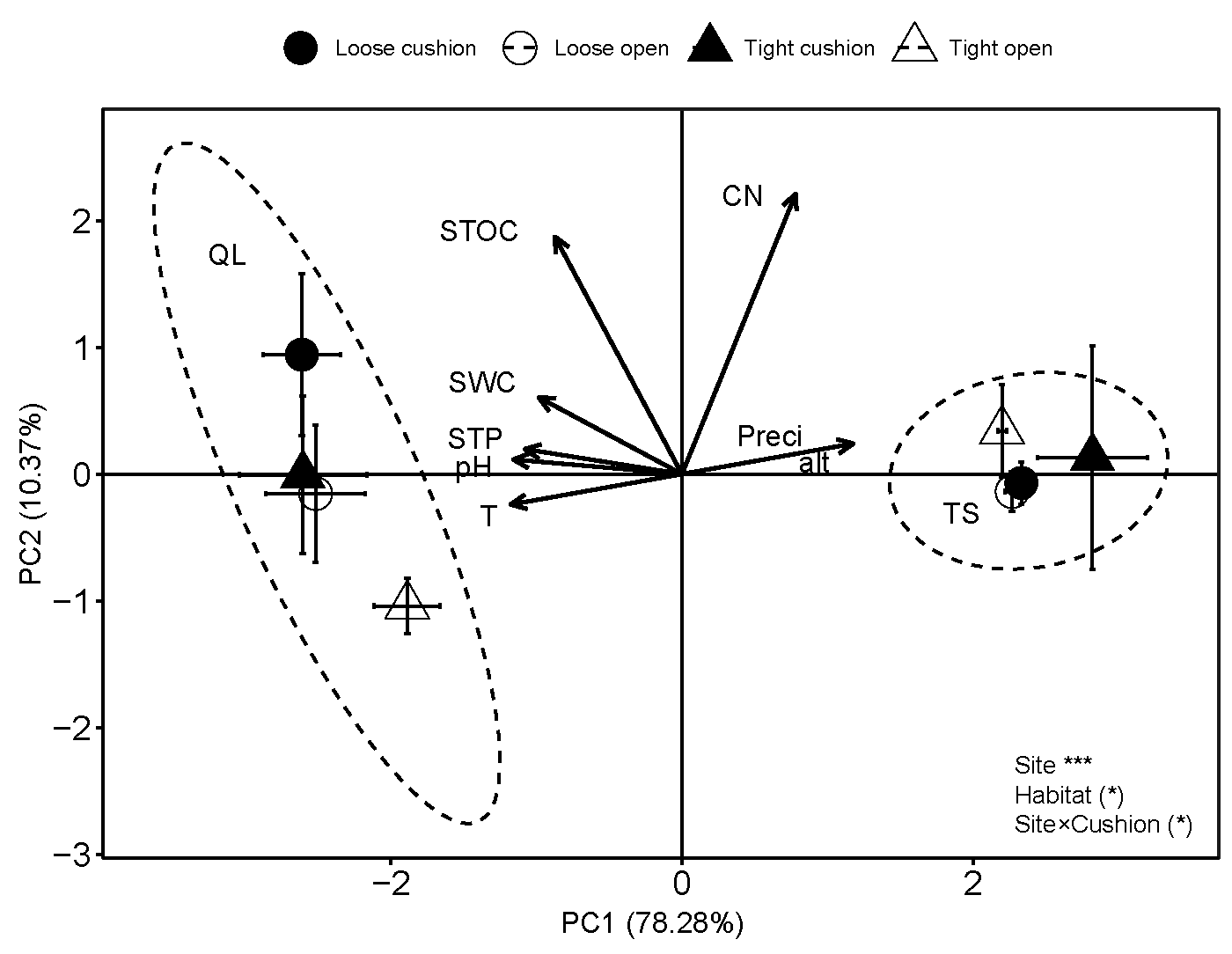

Supplement: Supplementary file 4 [file Image_3.tif]
